# Committing to anti-racism reforms? Three critical building blocks for global health organizations

**DOI:** 10.1371/journal.pgph.0000653

**Published:** 2022-07-08

**Authors:** Mishal S. Khan, Angela Obasi, Rinki Deb, Serign Jawo Ceesay

**Affiliations:** 1 London School of Hygiene and Tropical Medicine, London, England; 2 Liverpool School of Tropical Medicine, Liverpool, England; 3 Co-Chair of the Race Equality Advisory Panel and BAME Staff Network, Liverpool School of Tropical Medicine, Liverpool, England; 4 Overseas Staff Representative on Governing Council Diversity & Inclusion Committee, London School of Hygiene and Tropical Medicine, London, England; PLOS: Public Library of Science, UNITED STATES

Independent reviews of racism in organisations working to improve global health and justice are piling up. In 2021, a report on abuse and discrimination at Doctors Without Borders was published [1]. In April 2022, Amnesty International UK reported that initial findings from their investigation suggested that the organisation “exhibits institutional racism” [2]. Two recent independent reviews highlighting pervasive attitudes, behaviours and processes that disadvantage minoritized individuals at two of the oldest and most renowned institutes of global health in the world–the Liverpool School of Tropical Medicine (LSTM) and the London School of Hygiene and Tropical Medicine (LSHTM)–therefore raise issues that are not unique to these institutions [3, 4]. Neither are the predicaments associated with moving ahead.

Leadership of numerous global health institutions have ostensibly committed to working towards becoming anti-racist. However, the task ahead is an enormous one that will require radical reforms, especially since global health institutions are neither global nor diverse [5]; for example, the 2022 Global Health 50/50 report highlights that approximately three-quarters of decisions-makers on governing boards of global health organisations are from high income countries, with 51% from the UK and US [6].

Having been closely involved in the ‘anti-racism and decolonisation’ journeys of LSHTM and LSTM, we lay out three critical building blocks that we believe are essential for meaningful reform organisations working in global health. Although written with global health academia in mind, we anticipate these would apply to the increasing number of organisations working in global health that are confronting similar issues.

## 1. Hand over (some, if not all) decision-making power to people with credibility and expertise

Institutional leadership acknowledging the attitudes, structures and culture underlying structural racism is an important first step, as is publicly committing to reforms. But after this initial step, it is essential for those who have been holding the reins to ‘lean out’ and allow decisions about strategic priorities and implementation to be driven by those who are trusted by affected groups and have a track-record of shaking up the status quo. While this might sound simple, it is understandably uncomfortable for some in leadership positions to accept that a core issue is a lack of trust that people in positions of power are able or willing to make deep reforms [7]. One can debate whether this lack of trust is warranted or not, but it is irrelevant to a great extent. When there is a clear trust and confidence deficit, it should be obvious that moving forward successfully requires a set of trusted people to step in. In the long-term, this is essential for success. In the short-term, this will demonstrate that there is a real desire for change.

It is also important to realise that having lived experience of racism, although potentially useful in shaping necessary reforms, does not alone equate to having the required expertise that is needed to reform institutions’ deep-rooted systems and culture. Indeed, there is a persisting failure to understand and invest in real expertise in anti-racism and decolonisation. Further, solutions made by people who have primarily worked in the same sector, for example academia, may be insufficiently bold in their transformation approaches, because we are all shaped by the structures that we have operated in.

## 2. Understand the difference between ‘Equity, Diversity and Inclusion’ strategies and ‘transformative anti-racism’

There is a widespread and dangerous misunderstanding that increasing the diversity of staff or students, and including more minoritized individuals in consultations to inform strategic decisions, will sort out racism issues. Perhaps it is also a convenient misunderstanding to maintain. Most Equity, Diversity and Inclusion (EDI) plans do not address the root causes of individual and structural racism and are therefore inadequate. We are not scholars in anti-racism or decolonisation, and we sincerely acknowledge the importance of real expertise in this area, so here we simply highlight some fundamental flaws with many EDI practices that we have observed.

*Increased diversity*—it is not difficult, or sufficient, to fill up seats on a decision-making table with minoritised individuals who will support the status quo; bold challenge is core to the reforms needed. *Increased inclusion*–a common practice being highlighted as a positive step forward is ‘listening sessions’ with minoritized groups, in which the existing leadership retains the power to decide which comments are taken on board and to what extent; such inclusion activities therefore continue to centre the powerful and tend not to result in strategies that address core issues such as privilege and power dynamics.

Processes that support and reinforce cultural and organisational structures of marginalisation will–naturally–fail to achieve the deep-rooted changes needed. Focusing instead on power and privilege, to rebalance who gets to make decisions and who benefits from the systems in place, is a very different approach. It is abundantly evident that racism and colonialism are structural drivers of inequities in global health and development [8]. Indeed, structural racism has correctly been labelled a “global social epidemic” [9]. This makes a cross cutting transformative antiracism–essentially looking at power and privilege through the lens of racism—especially essential in global health. EDI does not do this, and this is why EDI is increasingly being seen as a tick-box or gloss-over exercise [10].

## 3. Reward the effort and risks involved in speaking out

The investment that academic institutions make for rooting out racist and colonial practices are to be congratulated. At the same time, one cannot ignore the advocacy by brave individuals who were instrumental in getting institutions to accept that there are serious issues to be confronted. For both LSTM and LSHTM, independent reviews are anticipated to be an essential first step in the path to transparent reform and rebuilding of trust. However, to make these reviews happen, members of volunteer run networks of staff and students had to speak out publicly about traumatic racism that they had experienced and highlight inadequacies in earlier responses to racism, being cognisant of the potential risk of reprisal. They also had to conduct months of lobbying to convince leadership that any review should be conducted by a fully independent reviewer rather than an internal “task force”. Further, once the principle of independent review was agreed, there was enormous work done to negotiate what constitutes independence and ensure that appropriate steps were undertaken to ensure independence. This lobbying and negotiation took substantial effort from minoritized individuals and their allies; with the former already facing challenges arising from cultures and processes that are hostile to them. This effort has both a value and a cost.

However, current assessments of academic excellence do not include metrics for furthering fairness and equity. This means these attributes essentially have no academic value and that the labour of promoting fairness also has no worth [11]. But the opportunity costs and adverse consequences for staff and students of colour who advocate for racial equity are potentially vast. It is past time that the effort and risks involved in pushing for justice and equity are recognised and rewarded. It is an essential step towards addressing the cultures of unfairness, aggression and bullying that are increasingly recognised as a threat to quality and conduct of science [12].

## But why do all of this?

Commitment statements and superficial changes are much easier than giving up power and resources, even for people who are sincere in their intentions. There needs to be a compelling reason for deep reforms [13]. And there is. The Covid pandemic has raised awareness and intolerance for the inequities and injustice in health–be this within organisations or collaborations or across countries. For example, there is demand from students for decolonisation of global health curricula and teaching–including to be taught by experts from the settings that are being discussed. Researchers are seeking to move ahead on better equity in global health partnerships and publishing [14]. Funders are increasingly examining the racial equity of their own practices and expecting Southern leadership or co-leadership of research grants [15]. This means that research institutions from the Global South will increasingly have the power to demand respect and fair treatment from their northern partners.

With this turning tide, institutions that are proactively engaging in fairer ways of working will be favoured by students, employees and funders, and international partners. Leaders with real vision will see that letting go of some power and resources, as part of a necessary evolutionary process, is better than being left behind.
